# Role of Polymer-Free Drug-Eluting Stents in Insulin-Dependent Diabetic Patients Undergoing Percutaneous Coronary Intervention: An Observational Study

**DOI:** 10.3390/jpm15120594

**Published:** 2025-12-03

**Authors:** Filippo Luca Gurgoglione, Davide Donelli, Marco Frazzetto, Luigi Vignali, Giorgio Benatti, Iacopo Tadonio, Andrea Denegri, Marco Covani, Mattia De Gregorio, Gabriella Dallaglio, Giampaolo Niccoli, Bernardo Cortese, Emilia Solinas

**Affiliations:** 1 Division of Cardiology, Parma University Hospital, 43126 Parma, Italy; filippoluca.gurgoglione@unipr.it (F.L.G.); davide.donelli@unipr.it (D.D.); itadonio@ao.pr.it (I.T.); adenegri@ao.pr.it (A.D.); marco.covani@unipr.it (M.C.); gabriella.dallaglio@unipr.it (G.D.);; 2 DCB Academy, 20136 Milano, Italy; marcofrazzetto7@gmail.com (M.F.);; 3 Harrington Heart & Vascular Institute, University Hospitals Cleveland Medical Center, Cleveland, OH 44106, USA; 4 Department of Medicine and Surgery, University of Parma, 43125 Parma, Italy

**Keywords:** diabetes mellitus, insulin, coronary artery disease, percutaneous coronary intervention, polymer-free stents, propensity matching analysis, prognosis, personalized PCI strategy

## Abstract

**Background/Objectives**: Diabetes mellitus (DM), especially insulin-dependent DM (IDDM), is strongly associated with adverse outcomes following percutaneous coronary intervention (PCI) failure. Polymer-free drug-eluting stents (PF-DESs) have emerged as a promising strategy to mitigate long-term coronary inflammation. This study aimed to evaluate the role of PF-DES, as compared to permanent-polymer DES (PP-DES) and biodegradable-polymer DES (BP-DES), in a real-world cohort of IDDM patients with obstructive coronary artery disease (CAD) undergoing PCI. **Methods**: IDDM patients with CAD who underwent PCI with DES at Parma University Hospital were divided into two study groups: PF-DES group vs. BP/PP-DES group. The primary endpoint was target vessel failure (TVF) at the 4-year follow-up. Survival analyses and propensity score matching (PSM) were performed to account for baseline differences. **Results**: A total of 170 IDDM patients with 215 treated lesions (31.6% PF-DES; 68.4% BP/PP-DES) were included. The PF-DES group experienced significantly lower rates of TVF (10.3% vs. 27.2%, *p* < 0.01, log rank *p* = 0.0072) compared with the BP/PP-DES group. PSM analysis confirmed the good clinical performance of PF-DES (HR 0.27, *p* < 0.01). **Conclusions**: In this PSM-based observational study, PF-DESs were associated with significantly lower rates of TVF compared with BP/PP-DESs in IDDM patients undergoing PCI for CAD. These suggest that PF-DES may represent a personalized PCI strategy for IDDM patients, with prognostic benefits that become increasingly pronounced as the clinical and anatomical risk profile worsens.

## 1. Introduction

Diabetes mellitus (DM) represents one of the strongest risk factors for coronary artery disease (CAD) [1], which continues to be the main cause of death in this population [2].

The introduction of drug-eluting stents (DESs) has markedly improved outcomes in patients undergoing percutaneous coronary intervention (PCI) [3]. Nevertheless, PCI failure, due to stent thrombosis and in-stent restenosis (ISR), remains a relevant issue [4,5], especially among patients with DM [6,7].

To overcome these challenges, technological progress in DES technology have led to the development of polymer-free DESs (PF-DESs) [8]. These stents elute antiproliferative drugs without the need for a polymer matrix, potentially reducing coronary inflammation [9]. However, most available PF-DESs feature thin stent struts, which might compromise radial strength and raise procedural concerns, particularly when treating complex or calcified coronary lesions [10].

While PF-DESs have been shown to attenuate late lumen loss compared with permanent-polymer (PP-DESs) and biodegradable-polymer DESs (BP-DESs) [11], their clinical efficacy remains uncertain. Previous randomized clinical trials (RCTs) have enrolled heterogeneous cohorts of patients with DM, yielding inconsistent results [12,13,14,15,16,17,18,19,20,21,22,23,24].

Insulin-dependent diabetes mellitus (IDDM) refers to a diabetic condition in which insulin therapy is required for patients who do not adequately respond to oral hypoglycemic agents, as well as for those who have developed microvascular or macrovascular complications [25]. This subset represents a particularly high-risk subset, characterized by more advanced CAD [25] and nearly double the risk of PCI failure compared with non-IDDM patients [26,27]. From a pathophysiological point of view, PF-DES may represent a tailored PCI approach for IDDM patents. However, evidence specifically addressing the role of PF-DES both in DM patients and in this specific population is limited.

In the present study, we aimed to evaluate the role of PF-DES compared with BP-DES and PP-DES in a real-world cohort of IDDM patients with CAD undergoing PCI.

## 2. Materials and Methods

Study Population

**Figure 1:**
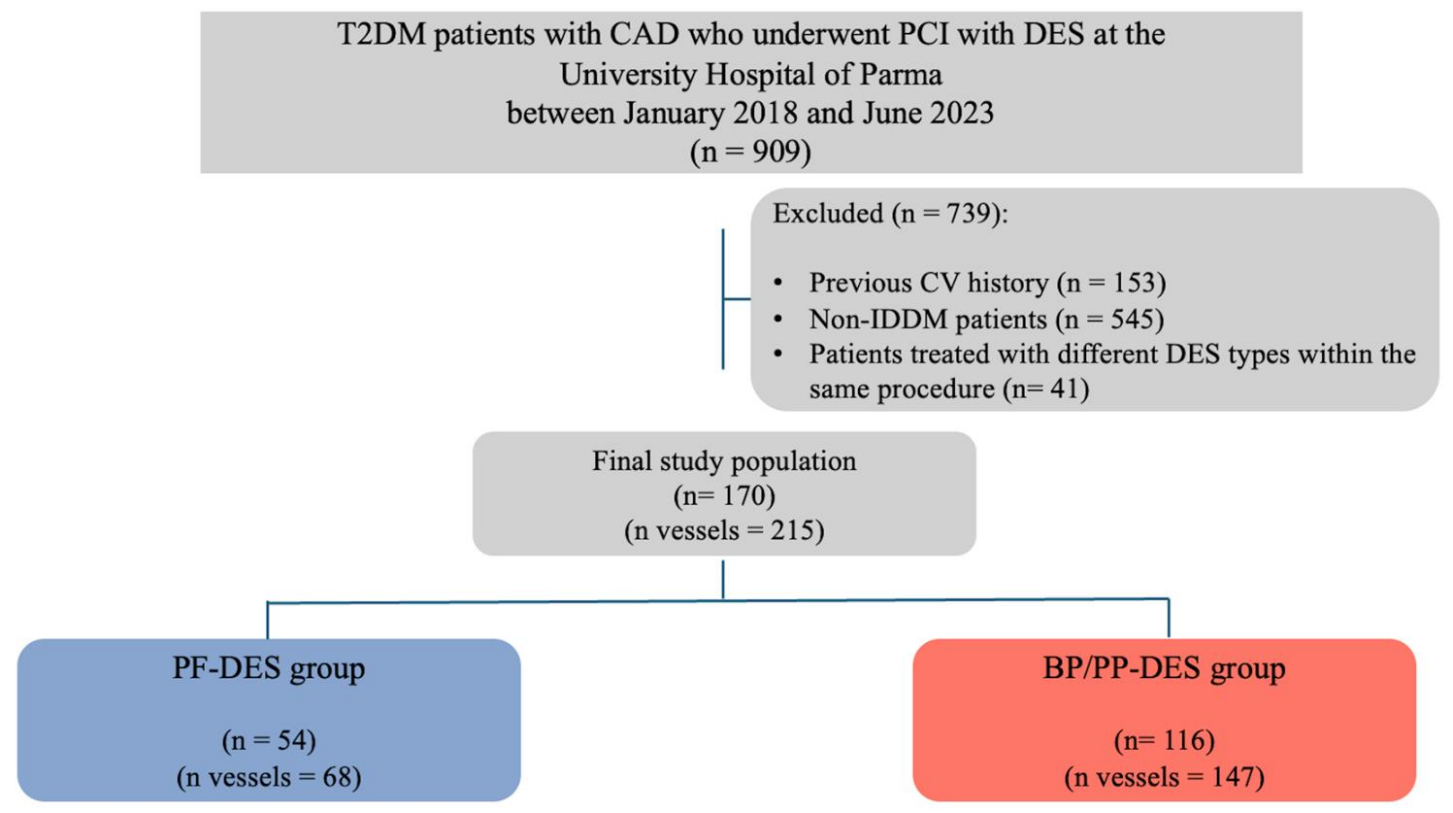
Study flow chart. Abbreviations: BP/PP-DES: biodegradable-polymer/permanent-polymer drug-eluting stent; CAD: coronary artery disease; CV: cardiovascular; DES: drug-eluting stent; IDDM: insulin-dependent diabetes mellitus; PCI: percutaneous coronary intervention; PF-DES: polymer-free drug-eluting stent; T2DM: type 2 diabetes mellitus.

This was a single-center observational study that included consecutive DM patients with CAD who underwent PCI with DES at the University Hospital of Parma between January 2018 and June 2023 (Figure 1). Both patients admitted for chronic coronary syndrome and those presenting with acute coronary syndrome (ACS) were eligible, according to the most recent European Society of Cardiology guidelines [28,29]. DM status was defined by a known clinical history of type 2 DM or diagnosis based on American Diabetes Association diagnostic criteria [30]. Baseline pharmacologic therapy at hospital admission was recorded for each patient.

Exclusion criteria included a prior cardiovascular history (defined as previous hospitalization for ACS or chronic coronary syndrome, prior coronary revascularization, heart failure, any valvular heart disease greater than mild, or any congenital heart disease).

From the initial cohort of 909 patients, only those with IDDM who underwent single- or multivessel PCI using the same DES strategy (PF-DES, BP-DES, or PP-DES) for all coronary lesions—either during the index procedure or in a staged approach—were selected. The final study population comprised 170 patients with a total of 215 treated lesions, stratified into two groups according to PCI/DES strategy: PF-DES vs. BP/PP-DES.

The study protocol was registered on the Open Science Framework (https://doi.org/10.17605/OSF.IO/DJ457), conducted in accordance with the Declaration of Helsinki, and approved by the Area Vasta Emilia Nord Ethical Committee on 5 April 2023 (73/2023/OSS/AOUPR). Written informed consent was obtained from all participants before enrollment.

Coronary angiography and PCI details

Coronary angiography was performed via either the radial or femoral artery approach. To ensure complete visualization of the coronary tree, at least two perpendicular projections were acquired for the right coronary artery and four for the left coronary artery. The anatomical complexity of CAD was assessed using the Synergy between Percutaneous Coronary Intervention with TAXUS and Cardiac Surgery (SYNTAX) score [31] and the SYNTAX score II [32]. In addition, the modified American College of Cardiology/American Heart Association (ACC/AHA) ABC classification [33] was applied for morphological lesion characterization, stratifying all coronary lesions into two categories: non-complex (ACC/AHA A–B1) and complex (ACC/AHA B2–C).

PCI procedures were performed according to current standards of care [34], with the choice of DES left to the operator’s discretion. In line with the real-world design of this study, the following devices were available in the catheterization laboratory of Parma University Hospital and were employed accordingly: PF-DES (Cre8™ (Alvimedica, Saluggia, Italy)), Coroflex™ (B. Braun, Melsungen, Germany), BioFreedom™ (Biosensors, Morges, Switzerland), BP-DES (Synergy™ (Boston Scientific, Marlborough, MA, USA)), Cruz™ (Sahajanand Medical Technologies, Surat, India), and PP-DES (Xience™ (Abbott, Santa Clara, CA, USA)), Onyx™ (Medtronic, Minneapolis, MN, USA), Promus™ (Boston Scientific, Marlborough, MA, USA). A detailed description of the technical characteristics of each DES is provided in Supplementary Table S1.

Follow-up and clinical assessment

Clinical follow-up was conducted through outpatient clinic visits or telephone interviews at 3–12 months after PCI and annually thereafter for up to 4 years. The primary endpoint was target vessel failure (TVF), defined as a composite of cardiac death, target vessel myocardial infarction (TVMI), and target lesion revascularization (TLR). Cardiac death was defined as sudden death or death preceded by typical chest pain within 24 h. TVMI was defined as myocardial infarction with evidence of myocardial necrosis in the vascular territory of the previously treated target vessel. TLR was defined as any repeat revascularization procedure (percutaneous or surgical) of the original target lesion, including the stented segment and within 5 mm proximal or distal to the stent margins [35].

Statistical analysis

Baseline characteristics were summarized at both patient and lesion levels using descriptive statistics and compared between study groups. Continuous variables were analyzed with independent two-sample t-tests if normally distributed or with the Wilcoxon rank-sum test if skewed. Categorical variables were compared using the chi-square test. Time-to-event outcomes were assessed with Kaplan–Meier survival analysis. Propensity score matching (PSM) was applied to balance observed confounders between groups. Post-matching balance was evaluated using standardized mean differences, with values <0.1 indicating adequate balance (Supplementary Figure S1). After PSM, weighted Cox proportional hazards regression was used to estimate hazard ratios (HRs) for TVF. Post hoc analyses were performed to compare clinical outcomes between the two groups within specific subgroups of interest, such as patients with ACS and those with complex target lesions. Statistical analyses were performed with R Studio version 4.4.1, and a two-tailed *p*-value <0.05 was considered statistically significant.

## 3. Results

Clinical characteristics

A total of 170 patients were enrolled (mean age 69.4 ± 11.6 years; 79 (46.5%) men), of whom 130 (76.5%) presented with ACS. The majority (116 (68.2%)) received BP-/PP-DES, while approximately one-third (54 (31.8%)) underwent PCI with PF-DES. The cohort was characterized by a high prevalence of traditional cardiovascular risk factors: smoking in 51.8%, hypertension in 86.5%, dyslipidemia in 78.8%, and chronic kidney disease in 27.1%. No significant differences in clinical, laboratory, or echocardiographic features were observed between the two groups (Table 1).

Angiographic and PCI data

Most patients (129 (75.9%)) had single-vessel CAD, with the majority of lesions located in the left anterior descending artery (108 (63.5%)). The mean SYNTAX score was 13.0 ± 6.1, and the mean SYNTAX score II was 38.2 ± 12.2. Procedural characteristics were comparable between groups (Table 2). In total, 215 lesions were treated, with 68 (31.6%) receiving PF-DES and 147 (68.4%) receiving BP-/PP-DES. Of these, 82 (38.1%) were classified as complex (ACC/AHA B2–C). The most frequently implanted DES was Coroflex™ (50 (23.3%)) in the PF-DES group and Xience™ (88 (40.9%)) in the BP-/PP-DES group. The mean number of stents per patient was 1.6 ± 0.7, with a mean total stent length of 38.1 ± 17.4 mm.

Clinical outcomes

**Figure 2:**
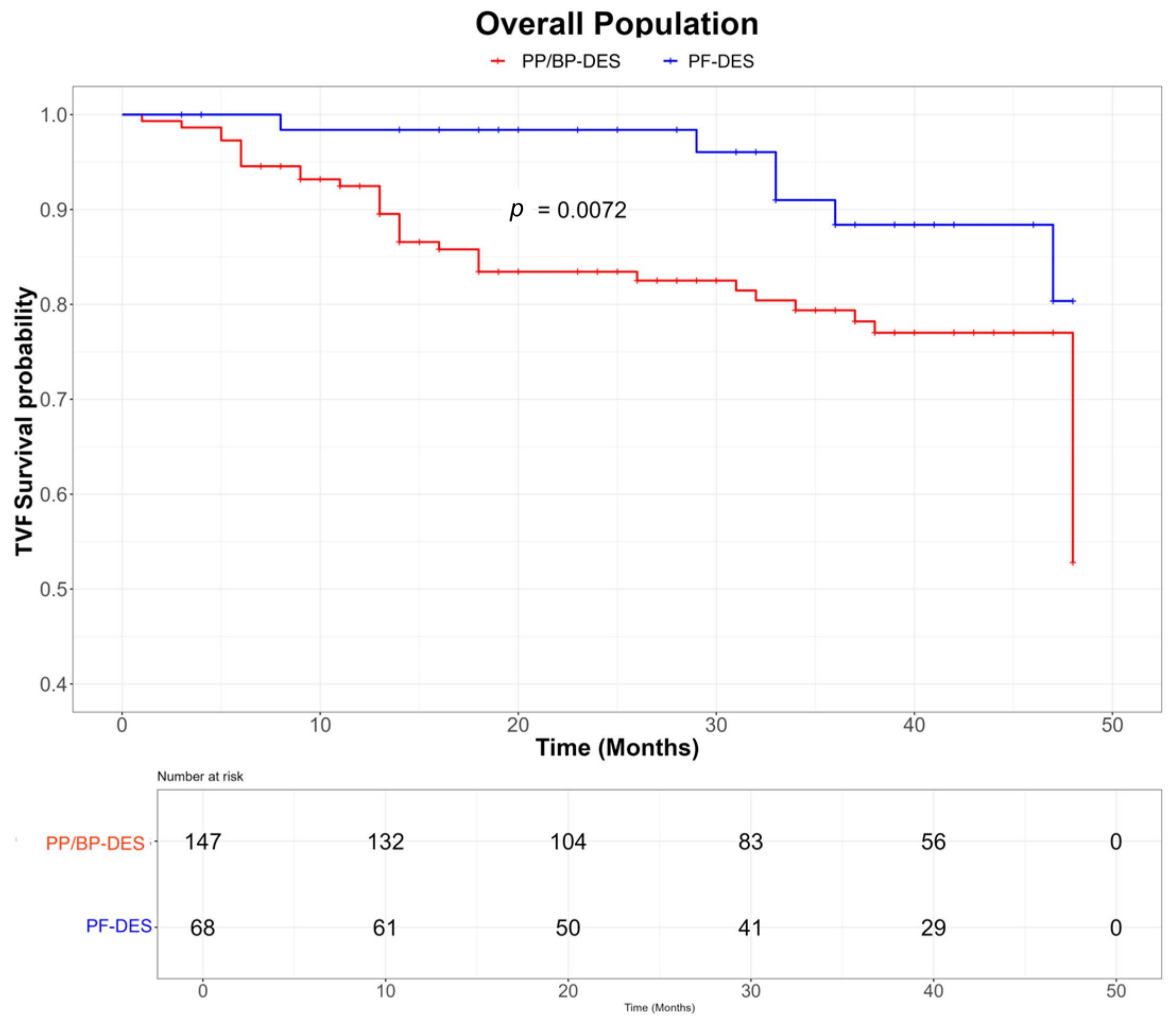
KM curves for TVF in the overall population according to PF-DES vs. PP/BP-DES groups. Abbreviations: PF-DES: polymer-free drug-eluting stent; PP/BP-DES: permanent-polymer/biodegradable-polymer drug-eluting stent; TVF: target vessel failure.

**Figure 3:**
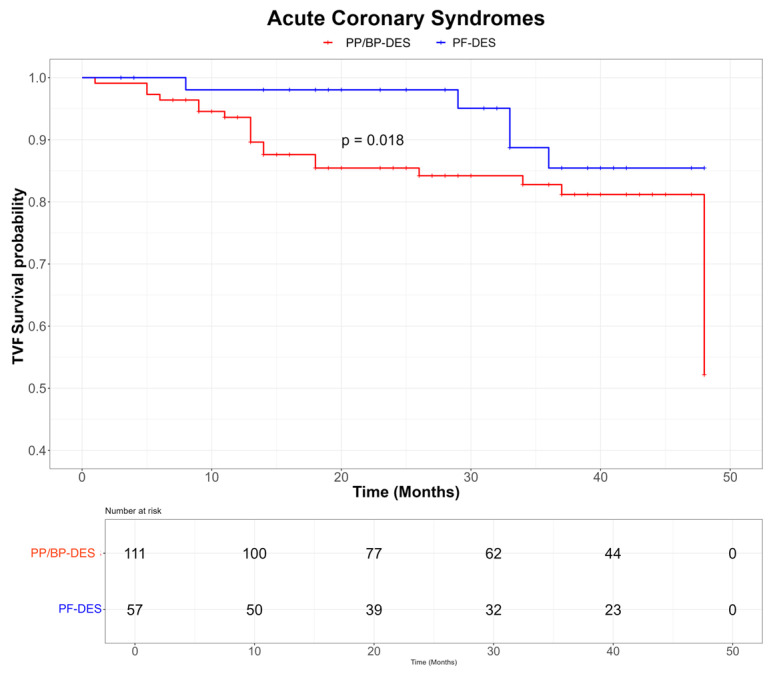
KM curves for TVF in the subgroup with ACS according to PF-DES vs. PP/BP-DES groups. Abbreviations: PF-DES: polymer-free drug-eluting stent; PP/BP-DES: permanent-polymer/biodegradable-polymer drug-eluting stent; TVF: target vessel failure.

**Figure 4:**
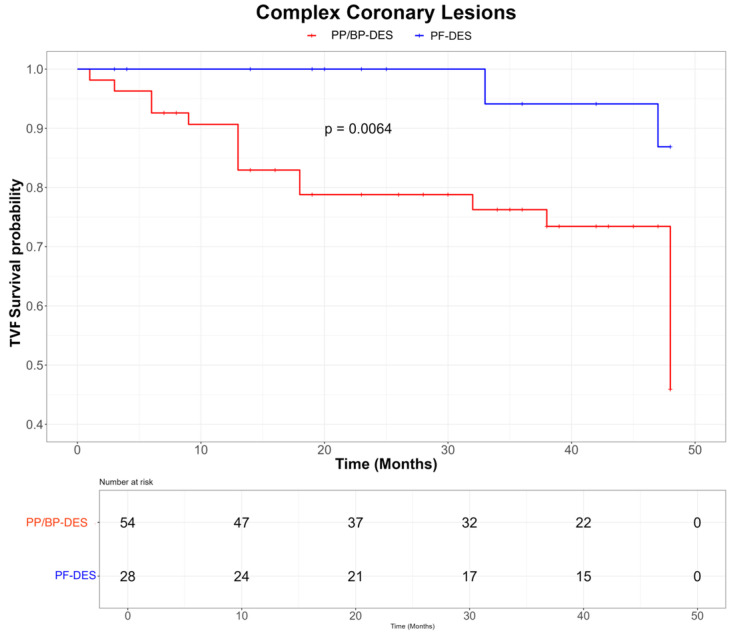
KM curves for TVF in the subgroup with complex coronary lesions according to PF-DES vs. PP/BP-DES groups. Abbreviations: PF-DES: polymer-free drug-eluting stent; PP/BP-DES: permanent-polymer/biodegradable-polymer drug-eluting stent; TVF: target vessel failure.

At the 4-year follow-up, the PF-DES group showed a significantly lower incidence of TVF compared with the BP-/PP-DES group (10.3% vs. 27.2%, *p* < 0.01), primarily driven by reduced rates of TVMI (1.5% vs. 8.1%, *p* = 0.01) and TLR (2.9% vs. 17.9%, *p* < 0.01) (Table 2). Two cases of stent thrombosis were observed in the BP-/PP-DES group, with no events reported in the PF-DES group. Kaplan–Meier curves demonstrated lower rates of TVF in the PF-DES group compared with the BP-/PP-DES group in the overall population (log-rank *p* = 0.0072; Figure 2) as well as in patients presenting with ACS (log-rank *p* = 0.018; Figure 3) and in those with complex lesions (log-rank *p* = 0.0064; Figure 4). Finally, a sensitivity analysis including only lesions treated with the most frequently used stents (Coroflex™ in the PF-DES group and Cruz™, Synergy™, and Xience™ in the BP-/PP-DES group) confirmed the lower incidence of TVF in the PF-DES group compared with the BP-/PP-DES group (10.0% vs. 24.6%, *p* = 0.048).

The PSM model included seven predictors, achieving a very good propensity balance (Supplementary Figure S1). Cox proportional hazards analysis demonstrated a significantly lower risk of TVF with PF-DES compared to BP-/PP-DES (HR 0.27, 95% CI 0.11–0.65, *p* < 0.01). The benefit of PF-DES was consistent in patients with ACS (HR 0.20, 95% CI 0.07–0.55, *p* < 0.01) and in those with chronic coronary syndrome (HR 0.12, 95% CI 0.02–0.93, *p* = 0.01) and complex lesions (HR 0.28, 95% CI 0.05–0.53, *p* = 0.03).

## 4. Discussion

The principal finding of this observational study is that revascularization with PF-DES, compared with BP-/PP-DES, was associated with significantly lower rates of the composite endpoint of TVF at the 4-year follow-up (10.3% vs. 27.2%, *p* < 0.01) in a real-world cohort of patients with IDDM and obstructive CAD. This evidence may help to tailor PCI strategies for patients with IDDM. Insulin-treated diabetic patients represent a particularly high-risk subgroup prone to PCI failure [6,7,25]. The interplay of endothelial dysfunction, chronic low-grade inflammation, and a prothrombotic milieu is more pronounced in IDDM, contributing to a two-fold higher risk of cardiac death and a three-fold higher risk of stent thrombosis compared with non-IDDM patients [26,27,36].

A subanalysis of the landmark ReCre8 trial first suggested a potential advantage of PF-DES over PP-DES in a small cohort of 95 IDDM patients at 12 months [37]. This study was limited by a relatively small sample size (96 IDDM patients), a direct comparison between only two specific devices (the polymer-free amphilimus-eluting stent (Cre8, Alvimedica, Istanbul, Turkey) and the permanent-polymer DES Resolute Integrity (Medtronic Vascular, Santa Rosa, CA, USA)), and a relatively short follow-up period [37]. Our study further extends these findings by including a larger cohort of IDDM patients with CAD undergoing PCI, mostly admitted for ACS, with a longer follow-up period. Our findings confirm the favorable clinical performance of PF-DES in IDDM patients undergoing PCI, although the results are limited by the single-center, retrospective design of the study and the relatively modest sample size.

Although mechanistic insights cannot be directly derived from our study, prior evidence have shown that the absence of a polymer matrix in PF-DES may reduce in-stent late lumen loss by mitigating polymer-related chronic inflammation, delayed vascular healing, and accelerated neoatherosclerosis [9,11]. The divergence of Kaplan–Meier curves for TVF observed after 6 months in our analysis further aligns with this pathophysiological rationale.

A notable result is that the good clinical performance of PF-DES was particularly evident among patients with ACS and those with complex coronary lesions. ACS is characterized by widespread pan-coronary vulnerability, driven by sustained vascular inflammation, and is associated with nearly a two-fold increased risk of PCI failure compared with stable CAD [38,39]. Similarly, PCI of complex lesions, especially bifurcations and heavily calcified plaques, remains associated with higher rates of late stent failure, frequently due to under-expansion or malapposition [40,41]. In this context, the intrinsic properties of PF-DES may mitigate these mechanisms, translating into improved long-term outcomes. Taken together, these findings suggest that the pathophysiological advantages of PF-DES may offer particular prognostic benefits in high-risk IDDM patients, especially in those presenting with ACS or requiring treatment of complex coronary anatomy. From a personalized medicine perspective, PF-DES might therefore be considered as a preferred PCI strategy in IDDM patients to reduce the risk of PCI failure during follow-up.

IDDM patients represent nearly one quarter of all individuals with diabetes undergoing PCI and experience substantially worse outcomes, including a 1.7-fold higher risk of mortality and stent thrombosis, and a 1.4-fold higher risk of MI and TLR compared with non-IDDM patients [26]. In this vulnerable population, our results indicate that a personalized approach using PF-DES may help mitigate adverse events during follow-up by reducing coronary inflammation and the pro-thrombotic milieu [21,22,24]. These prognostic advantages appear to be particularly relevant in patients with more severe clinical presentation (i.e., ACS) and complex coronary anatomy.

In summary, our data suggest that PF-DES may represent a personalized PCI strategy for IDDM patients, with prognostic benefits that become increasingly pronounced as the clinical and anatomical risk profile worsens.

Limitations

Several limitations of this analysis should be acknowledged. First, the observational design inherently carries the risk of residual confounding, and factors such as operator decision-making, socioeconomic status, and adherence to antiplatelet therapy were not accounted for in the analysis. Despite the applied methodological adjustments, this limitation precludes definitive causal inferences. Second, the relatively small sample size and limited number of primary endpoint events may have reduced the statistical power of the study. In particular, subgroup analyses included a limited number of patients, and the results should be considered hypothesis-generating rather than conclusive. A post hoc power analysis based on the observed event rates indicated an estimated statistical power of approximately 80% for TVF occurrence in the overall population, 88% in the ACS subgroup, and 53% in the subgroup with complex target lesions. Third, the real-world nature of the investigation, involving multiple DES platforms with differences in strut thickness, design, and antiproliferative drugs, may have introduced treatment-related heterogeneity. Fourth, the lack of a core laboratory for angiographic and procedural assessment could have led to misclassification or measurement bias. Finally, other PCI strategies, such as the use of drug-coated balloons (DCBs), which have shown benefits in the revascularization of patients with diabetes [42,43,44,45] and complex CAD [46,47,48], were not evaluated in our study. Current evidence indicates that DCBs and DESs provide similar results in diabetic patients in both de novo lesions [44] and in-stent restenosis [45], with a promising trend suggesting a potential advantage of DCBs in reducing TVR in de novo coronary lesions [44]. From a pathophysiological perspective, DCB angioplasty may mitigate coronary inflammation, promote earlier vessel healing, and facilitate positive vascular remodeling. These mechanisms could be particularly beneficial in patients with diabetes, who are often characterized by diffuse and complex coronary artery disease [24,25,44,45,46]. Future research is warranted to compare this approach with PF-DES and PP-DES.

## 5. Conclusions

In this propensity score-matched observational study, PF-DESs were associated with significantly lower rates of TVF compared with BP-/PP-DESs in IDDM patients with CAD. The good clinical performance of PF-DES appeared particularly pronounced in patients presenting with ACS and in those with complex coronary anatomy. This study may help to individualize PCI strategies in patients with IDDM. Our data suggest that PF-DES may represent a personalized PCI strategy for IDDM patients, with prognostic benefits that become increasingly pronounced as the clinical and anatomical risk profile worsens.

Dedicated randomized controlled trials are warranted to confirm these findings and to define the role of PF-DES in this high-risk population.

## Figures and Tables

**Table 1:** Baseline clinical, laboratory and angiographic features in the overall population and according to PF-DES vs. BP/PP-DES groups.

Characteristics	Overall Population (*n =* 170)	PF-DES Group (*n =* 54)	BP/PP-DES Group (*n =* 116)	*p* Value
Clinical characteristics				
Age [years, mean (SD)]	69.4 (11.6)	69.9 (12.2)	69.1 (11.3)	0.48
Male sex [*n*, (%)]	79 (46.5)	24 (44.4)	55 (47.4)	0.59
Hypertension [*n*, (%)]	147 (86.5)	48 (88.9)	99 (85.3)	0.48
Smoking habit [*n*, (%)]	88 (51.8)	31 (57.4)	57 (49.1)	0.44
Dyslipidaemia [*n*, (%)]	134 (78.8)	39 (72.2)	95 (81.9)	0.15
BMI [mean (SD)]	28.1 (4.7)	28.1 (4.7)	28.1 (4.7)	0.66
Family history of CAD [*n*, (%)]	139 (81.8)	11 (20.4)	20 (17.2)	0.54
CKD (eGFR < 60 mL/min per 1.73 m^2^) [*n*, (%)]	46 (27.1)	19 (35.2)	27 (23.3)	0.10
AF [*n*, (%)]	16 (9.4)	6 (11.1)	10 (8.6)	0.57
History of stroke [*n*, (%)]	6 (3.5)	1 (1.9)	5 (4.3)	0.50
Clinical presentation [*n*, (%)]				0.32
CCS	40 (23.5)	9 (16.7)	31 (26.7)	
Unstable angina	7 (4.1)	2 (3.7)	5 (4.3)	
NSTEMI	87 (51.2)	29 (53.7)	58 (50.0)	
STEMI	36 (21.2)	14 (25.9)	22 (19.0)	
Laboratory data [median (IQR)]				
Hb (g/dL) [mean (SD)]	13.0 (1.8)	13.1 (1.8)	12.9 (1.8)	0.39
PLT (U/μL) [mean (SD)]	235.782 (71.015)	235.592 (69.905)	235.870 (71.827)	0.68
WBC (U/μL) [mean (SD)]	9.029 (3.759)	8.772 (2.711)	9.148 (4.163)	0.38
Glycaemia (mg/dL) [mean (SD)]	159.18 (72.62)	165.30 (67.06)	156.33 (75.17)	0.32
HbA1c (mmol/mol) [mean (SD)]	64.32 (19.76)	64.78 (19.46)	64.10 (19.98)	0.58
Total Cholesterol (mg/dL) [mean (SD)]	166.91 (52.27)	166.80 (53.58)	166.96 (51.88)	0.68
LDL Cholesterol (mg/dL) [mean (SD)]	93.54 (41.46)	94.19 (41.92)	93.24 (41.42)	0.62
HDL Cholesterol (mg/dL) [mean (SD)]	43.58 (11.27)	42.39 (11.26)	44.14 (11.28)	0.24
Serum creatinine on admission (mg/dL) [mean (SD)]	1.28 (1.29)	1.41 (1.29)	1.23 (1.28)	0.27
Echocardiographic data				
LVEF on admission (%) [mean (SD)]	47.26 (10.80)	48.09 (10.45)	46.88 (10.98)	0.35
Angiographic data				
SYNTAX Score (mean (SD))	13.05 (6.08)	13.22 (6.29)	12.97 (6.00)	0.55
SYNTAX 2 Score (mean (SD))	38.17 (12.24)	38.69 (11.69)	37.92 (12.53)	0.49
Number of Diseased Vessels (*n* (%))				0.53
1	129 (75.9)	42 (77.8)	87 (75.0)	
2	40 (23.5)	12 (22.2)	28 (24.1)	
3	1 (0.6)	0 (0.0)	1 (0.9)	
Left Main (*n* (%))	6 (3.5)	2 (3.7)	4 (3.4)	0.06
Left Anterior Descending Artery (*n* (%))	108 (63.5)	39 (72.2)	69 (59.5)	0.10
Left Circumflex Artery (*n* (%))	42 (24.7)	14 (25.9)	28 (24.1)	0.66
Right Coronary Artery (*n* (%))	55 (32.4)	12 (22.2)	43 (37.1)	0.06
Number of Lesions Treated (*n* (%))				0.55
1	126 (74.1)	40 (74.1)	86 (74.1)	
2	43 (25.3)	14 (25.9)	29 (25.0)	
3	1 (0.6)	0 (0.0)	1 (0.9)	
Therapy at discharge [*n*, (%)]				
Cardioaspirin	165 (97.1)	53 (98.1)	112 (96.6)	0.53
Clopidogrel	93 (54.7)	29 (53.7)	64 (55.2)	0.86
Ticagrelor	52 (30.6)	15 (27.8)	37 (31.9)	0.59
Prasugrel	23 (13.5)	9 (16.7)	14 (12.1)	0.44
OAC	16 (9.4)	4 (7.4)	12 (10.3)	0.52
β-blockers	160 (94.1)	53 (98.1)	107 (92.2)	0.06
CCBs	65 (38.2)	20 (37.0)	45 (38.8)	0.83
ACE-i/ARBs	120 (70.6)	36 (66.7)	84 (72.4)	0.46
Statin	154 (90.6)	48 (88.9)	106 (91.4)	0.62
Ezetimibe	25 (14.7)	7 (13.0)	18 (15.5)	0.64
Diuretics	71 (41.8)	23 (42.6)	48 (41.4)	0.88
Median DAPT duration (months) (median [IQR])	12 [6; 12]	12 [9; 12]	12 [6; 12]	0.96
Median TAT duration (months) (median [IQR])	1 [1; 3]	1 [1; 3]	1 [1; 3]	0.98

Legend to table: ACEi/ARBs angiotensin-converting enzyme inhibitors/angiotensin receptor blockers; AF: atrial fibrillation; BMI: body mass index; BP/PP-DES: biodegradable-polymer/permanent-polymer drug-eluting stent; CAD: coronary artery disease; CCBs: calcium-channel blockers; CCS: chronic coronary syndrome; CKD: chronic kidney disease; DAPT: dual antiplatelet therapy; GFR: glomerular filtration rate; Hb: hemoglobin; HbA1c: glycated hemoglobin; HDL: high-density lipoprotein; IQR: interquartile range; LDL: low-density lipoprotein; LVEF: low ventricular ejection fraction; NSTEMI: non-ST-elevation myocardial infarction; OACs: oral anticoagulants; PAD: peripheral arterial disease; PF-DES: polymer-free drug-eluting stent; PLT: platelets; SD: standard deviation; STEMI: ST-elevation myocardial infarction; SYNTAX: synergy between percutaneous coronary intervention with TAXUS and cardiac surgery; TAT: triple antiplatelet therapy; WBC: white blood count.

**Table 2:** Angiographic/PCI features and outcome data at the lesion level in the overall population and according to PF-DES vs. BP/PP-DES groups.

Characteristics	Overall Population (*n =* 215)	PF-DES Group (*n =* 68)	BP/PP-DES Group (*n =* 147)	*p* Value
Angiographic and PCI data ACC/AHA classification [*n*, (%)]				0.48
A	27 (12.6)	11 (16.2)	16 (47.4)	
B1	106 (49.3)	29 (42.6)	77 (10.9)	
B2	67 (31.2)	22 (32.3)	45 (30.6)	
C	15 (7.0)	6 (8.8)	9 (6.1)	
ACC-AHA complex lesions [*n*, (%)]	82 (38.1)	28 (41.2)	54 (36.7)	0.53
Calcific lesions [*n*, (%)]	80 (37.2)	28 (41.2)	52 (35.4)	0.42
Bifurcations [*n*, (%)]	66 (30.7)	24 (35.3)	42 (28.6)	0.33
Number of stents per patient [mean (SD)]	1.62 (0.69)	1.51 (0.61)	1.67 (0.71)	0.08
Total stent length [mean (SD)]	38.10 (17.24)	37.16 (16.57)	38.54 (17.58)	0.41
Implanted DES [*n*, (%)]				0.32
Biofreedom^TM^	5 (2.3)	5 (7.4)	0 (0.0)	
Coroflex^TM^	50 (23.3)	50 (73.5)	0 (0.0)	
Cre8^TM^	13 (6.0)	13 (19.1)	0 (0.0)	
Cruz^TM^	24 (11.2)	0 (0.0)	24 (16.3)	
Synergy^TM^	18 (8.4)	0 (0.0)	18 (12.2)	
Onyx^TM^	4 (1.9)	0 (0.0)	4 (2.7)	
Promus^TM^	13 (6.0)	0 (0.0)	13 (8.8)	
Xience^TM^	88 (40.9)	0 (0.0)	88 (59.9)	
Outcomes data				
TVF [*n*, (%)]	47 (21.9)	7 (10.3)	40 (27.2)	<0.01
Cardiac death [*n*, (%)]	11 (5.1)	5 (7.3)	6 (4.1)	0.36
TVMI [*n*, (%)]	13 (6.0)	1 (1.5)	12 (8.2)	0.01
TLR [*n*, (%)]	28 (13.0)	2 (2.9)	26 (17.9)	<0.01
ST [*n*, (%)]	2 (0.9)	0 (0.0)	2 (1.4)	0.34

Legend to table: ACC/AHA: American College of Cardiology/American Heart Association; BP/PP-DES: biodegradable-polymer/permanent-polymer drug-eluting stent; DES: drug-eluting stent; PCI: percutaneous coronary intervention; PF-DES: polymer-free drug-eluting stent; SD: standard deviation; ST: stent thrombosis; TLR: target lesion revascularization; TVF: target vessel failure; TVMI: target vessel myocardial infarction.

## Data Availability

The raw data supporting the conclusions of this article will be made available by the authors on request.

## Supplementary Materials

The following supporting information can be downloaded at: https://www.mdpi.com/article/10.3390/jpm15120594/s1, Figure S1: Love plot showing balancing of covariates after propensity matching analysis; Figure S2: KM curves for TVF in the overall population according to type of implanted DES; Table S1: Characteristics of implanted DES.

## Abbreviations

The following abbreviations are used in this manuscript:

ACC/AHA	American College of Cardiology/American Heart Association
ACS	acute coronary syndrome
BP-DES	biodegradable-polymer drug-eluting stent
CAD	coronary artery disease
DCB	drug-coated balloon
DES	drug-eluting stent
DM	diabetes mellitus
HRs	hazard ratios
IDDM	insulin-dependent diabetes mellitus
KM	Kaplan–Meier
PCI	percutaneous coronary intervention
PF-DES	polymer-free drug-eluting stent
PP-DES	permanent-polymer drug eluting stent
PSM	propensity score matching
RCTs	randomized controlled trials
TLR	target lesion revascularization
TVF	target vessel failure
TVMI	target vessel myocardial infarction
