# Supplementary material for: Role of Polymer-Free Drug-Eluting Stents in Insulin-Dependent Diabetic Patients Undergoing Percutaneous Coronary Intervention: An Observational Study
Source: J Pers Med. 2025 Dec 3;15(12):594. doi: 10.3390/jpm15120594 (PMC12733423; doi:10.3390/jpm15120594)
Supplement: Supplementary file 1 [file jpm-15-00594-s001.zip › mKGKQXi4y.pdf]

Table S1: Characteristics of implanted DES.

Type of DES	Manifacturer	Stent platform	Strut thickness	Antiproliferative drug	Kinetics of drug release
**PF-DES**					
Coroflex ™	B. Braun, Melsungen, Germany	Balloon-expandable stent manufactured from cobalt-chromium	55 μm (2.0 to 3.0 mm stents), 65 μm (3.5 to 4.0 mm stents)	Sirolimus (concentration of 1.2 μg/mm²) and probucol	80% of sirolimus is released within 30 days
Biofreedom ™	Biosensors Europe, Switzerland	Balloon-expandable stent manufactured from 316L stainless steel metal stent	120 μm	Biolimus A9,	1 month
Cre8 ™	CID S.p.A, Saluggia, Italy	Balloon-expandable stent manufactured from cobalt chromium L605 alloy	70 μm (2.0–2.25 mm stents), 80 μm (for the larger stents)	Sirolimus (90 μg/cm²) formulated with an amphiphilic carrier	70% of sirolimus is released within the first 30 days
**PP-DES**					
Xience ™	Abbott Laboratories, IL, USA	Balloon-expandable stent manufactured from cobalt chromium L605 alloy with a Fluorinated Copolymer	81 μm	Everolimus (average drug concentration of 100 μg/cm²)	more than 75% of the drug is released within the first 28 days
Onyx ™	Medtronic, Minneapolis, MN, USA	Balloon-expandable stent manufactured from a composite metal material, consisting of a cobalt-based alloy shell and a platinum–iridium alloy core	81 μm (for the 2.0–3.5 mm stents), 91 μm (for the 4.5–5.0 mm stents)	zotarolimus, 160 μg/cm²	180 days
Promus ™	Boston Scientific Corporation, Marlborough, USA	Balloon-expandable stent manufactured from platinum chromium	81 μm	Everolimus (1 μg of per mm²)	80% of the drug within 30 days
**BP-DES**					
Synergy ™	Boston Scientific Corporation, Marlborough, USA	Balloon-expandable stent made from a platinum chromium alloy along with a bioabsorbable polymer, poly-D,Llactide-co-glycolide	74 μm (2.25 mm to 2.75 stents), 79 μm (3.00mm to 3.50 mm stents), 81 μm (4.00 mm stents)	Everolimus (1 μg of per mm2)	**3-month release time**
Cruz ™	Sahajanand Medical Technologies, Surat, India	Balloon-expandable stent manifactured from cobalt-chromium alloy and a biodegradable polymer	60 μm	Sirolimus (concentration of 1.4 μg/mm²⁾	70% of the drug is released within 7 days

Table legend: BP-DES: biodegradable-polymer drug-eluting stents, PF-DES: polymer-free drug-eluting stents, PP-DES: permanent-polymer drug eluting stents

Figure S1: Love plot showing balancing of covariates after propensity matching analysis.

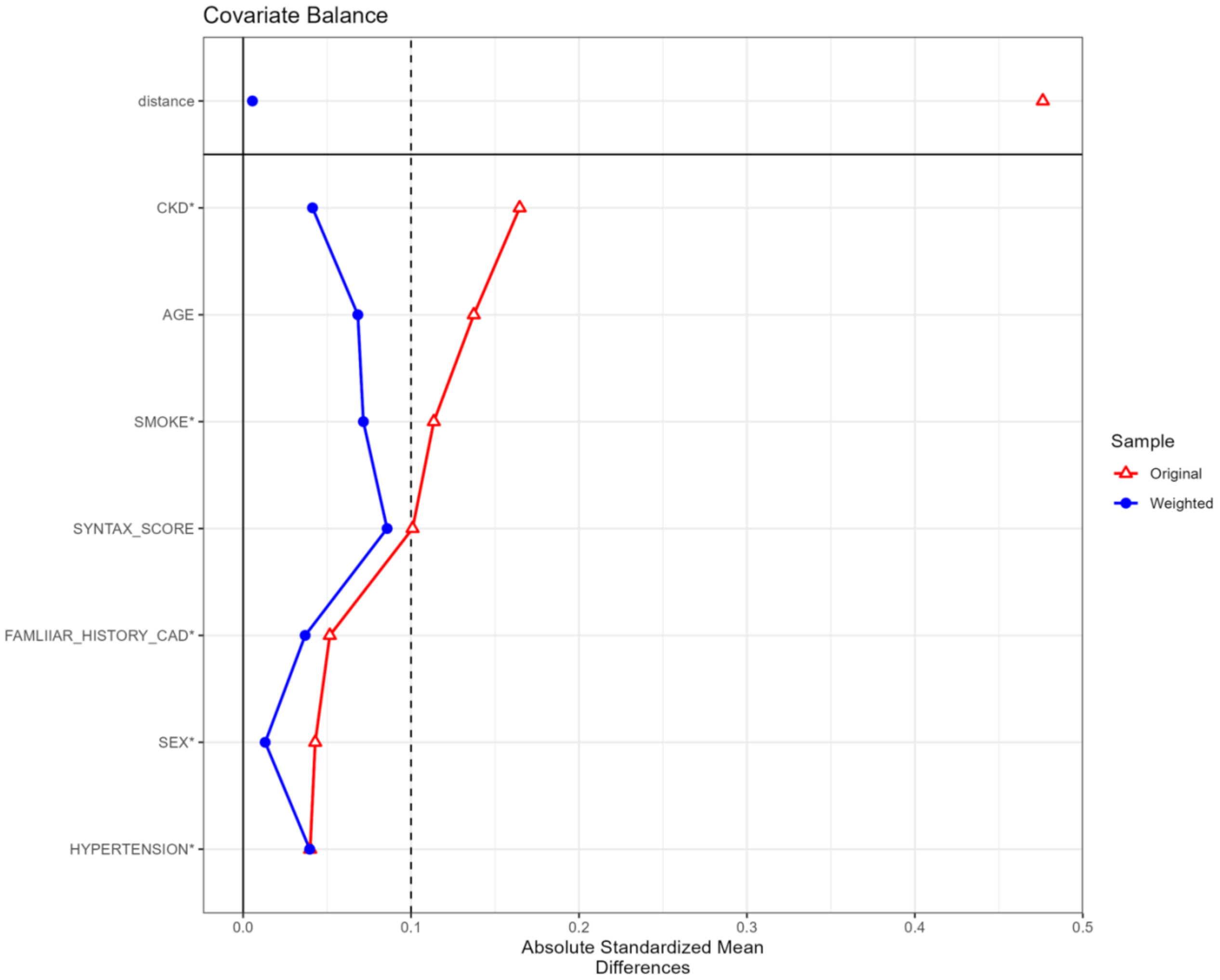

Abbreviations: CAD: coronary artery disease; CKD: Chronic kidney disease; SYNTAX: Synergy between Percutaneous Coronary Intervention with TAXUS and Cardiac Surgery
